# Host cell factors stimulate HIV-1 transcription by antagonizing substrate-binding function of Siah1 ubiquitin ligase to stabilize transcription elongation factor ELL2

**DOI:** 10.1093/nar/gkaa461

**Published:** 2020-06-01

**Authors:** Jun Wu, Yuhua Xue, Xiang Gao, Qiang Zhou

**Affiliations:** Department of Molecular and Cell Biology, University of California, Berkeley, CA 94720, USA; School of Pharmaceutical Sciences, Xiamen University, Xiamen, Fujian 361005, China; School of Pharmaceutical Sciences, Xiamen University, Xiamen, Fujian 361005, China; Department of Molecular and Cell Biology, University of California, Berkeley, CA 94720, USA

## Abstract

The Siah1 and Siah2 ubiquitin ligases are implicated in diverse biological processes ranging from cellular stress responses, signaling to transcriptional regulation. A key substrate of Siah1 is ELL2, which undergoes proteolysis upon polyubiquitination. ELL2 stimulates transcriptional elongation and is a subunit of the Super Elongation Complex (SEC) essential for HIV-1 transactivation. Previously, multiple transcriptional and post-translational mechanisms are reported to control Siah's expression and activity. Here we show that the activity of Siah1/2 can also be suppressed by host cell factor 1 (HCF1), and the hitherto poorly characterized HCF2, which themselves are not degraded but can bind and block the substrate-binding domain (SBD) of Siah1/2 to prevent their autoubiquitination and trans-ubiquitination of downstream targets including ELL2. This effect stabilizes ELL2 and enhances the ELL2-SEC formation for robust HIV-1 transactivation. Thus, our study not only identifies HCF1/2 as novel activators of HIV-1 transcription through inhibiting Siah1 to stabilize ELL2, but also reveals the SBD of Siah1/2 as a previously unrecognized new target for HCF1/2 to exert this inhibition.

## INTRODUCTION

Promoter-proximal pausing of freshly initiated RNA polymerase II (Pol II) on integrated HIV proviral DNA is a major rate-limiting step to restrict viral transcription (1,2). To overcome this restriction, the HIV-1-encoded Tat protein binds and recruits the human Super Elongation Complex (SEC) to paused Pol II through forming a multi-component complex on the TAR RNA element, a stem-loop structure located at the 5′ end of all nascent HIV-1 transcripts (2–5).

A SEC contains both P-TEFb and ELL2, two powerful elongation stimulatory factors that work by different mechanisms. Consisting of CDK9 and cyclin T1 (CycT1), P-TEFb phosphorylates the serine residues within the Pol II carboxy-terminal domain (CTD) as well as two negative elongation factors NELF and DSIF to antagonize their inhibitory actions (2). ELL2, on the other hand, can directly stimulate the processivity of Pol II by suppressing transient pausing, an intrinsic behavior of the polymerase. It has been shown that P-TEFb alone supports Tat-transactivation only mildly, whereas the combination of P-TEFb and ELL2 in a complete and functional SEC enhances the Tat activity by another 9-fold (6). Thus, upon recruitment by Tat and TAR to the HIV-1 promoter as integral components of a SEC, P-TEFb and ELL2 can synergistically activate Pol II elongation along the HIV-1 template to produce the full-length viral transcripts (2–5).

Among all the SEC subunits, ELL2 is stoichiometrically limiting and uniquely regulated at the level of protein stability (3). When it is bound by AFF4, which serves as a scaffolding molecule to organize the formation of the SEC (3,7), ELL2 becomes protected and stabilized (3). However, the free and freshly synthesized ELL2 protein can be quickly degraded by the proteasome upon polyubiquitination by the RING domain protein Siah1, which acts as an E3 ubiquitin ligase for ELL2 (8). In addition to ELL2, Siah1 and its homolog Siah2 collectively ubiquitinate more than 30 substrates including themselves, modulating a diverse array of biological processes that are involved in cellular stress responses, DNA damage control, transcription, cellular senescence, cancer- and neuro-associated functions (9).

The cellular transcriptional coactivator HCF1 (host cell factor 1), a component of several histone modification complexes, including histone demethylases and histone H3K4 methyltransferases, has been shown to play critical roles in regulating cell-cycle-dependent transcription (10) and maintenance of stem cell pluripotency (11). Besides its effects on cellular gene transcription, HCF1 has also been demonstrated as a host coactivator that promotes the initiation of transcription of the herpes simplex virus (HSV) Immediate Early (IE) genes. HCF1 performs this function by binding to multiple transcription factors, including the viral IE activator VP16, in the IE enhancer complexes and modulating the chromatin assembled on these genes (12).

In addition to HCF1’s role in IE transcriptional initiation, a recent study has revealed a surprising link between this coactivator and key components of the cellular transcriptional elongation machinery that include the SEC, the PAF complex and negative elongation factors DSIF and NELF (13). In particular, the SEC is shown as critical to drive IE gene expression, which is regulated by promoter-proximal pausing of Pol II, and productive infection, although exactly how the HCF1-SEC physical interaction contributes to this process awaits further investigation (13).

In light of the well-known function of the SEC in mediating HIV-1 Tat-transactivation and the recently revealed interaction between SEC and HCF1, we decided to investigate whether HCF1 also controls HIV-1 transcriptional elongation, and if so, whether its interaction with the SEC is involved. The results presented here indicate that both HCF1 and its homolog HCF2 are required for efficient HIV-1 elongation and that their endogenous levels are insufficient to support robust Tat-dependent HIV-1 transactivation. Importantly, HCF1 and HCF2 exert this effect by stabilizing ELL2 and promoting formation of the ELL2-containing SEC. Mechanistically, HCF1 and HCF2 are shown to antagonize the E3 ligase activity of Siah1 and Siah2 through binding and blocking the substrate-binding domain (SBD) of the two E3s. As a result of this, Siah1/2 fail to recognize their substrates including ELL2, TRAF2, β-Catenin and themselves, leading to their stabilization.

In the past, a variety of transcriptional and post-translational mechanisms have been shown to control the level and activity of the Siah ligases (9). Importantly, our present study has revealed the first example of a direct Siah inhibitor in HCF1 or HCF2, which are not degraded but can jam the substrate-binding function of the E3 ligases. In the case of HIV-1, this allows HCF1/2 to stimulate viral transcriptional elongation through interfering with Siah1’s catalytic activity, which in turn stabilizes ELL2 and enhances the ELL2-SEC formation.

## MATERIALS AND METHODS

### Antibodies and cDNAs

The antibodies against HCF1 (sc-390950), HCF2 (sc-393250), Cyclin T1 (H-245), TRAF2 (F-2) and β-Catenin (E-5) were purchased from Santa Cruz Biotechnology. The anti-AFF4 (ab57077) antibody was purchased from Abcam. The anti-AFF1 (A302-344A), -ENL (A302-268A), -AF9 (A300-595A), -ELL2 (A302-505A) and -ELL1 (A301-645A) antibodies were purchased from Bethyl Laboratories. The monoclonal antibodies against HA (3F10) and Flag (M2) were from Roche and Sigma-Aldrich, respectively. The antibodies against CDK9, LARP7 and Brd4 were generated in our own laboratory and have been described previously (14,15).

The HCF1 cDNA (pCGN-HCF-1; plasmid #53309) with an HA-Tag at the N-terminus and a Myc tag at the C-terminus was purchased from Addgene. The HCF2 cDNA (BC033799) was purchased from transOMIC technologies. The HCF2 cDNA was cloned into expression vectors N-Flag-PRK5M and N-HA-PRK5M, respectively.

### Generation of HCF1 or HCF2 knockdown (KD) cells

The most effective short hairpin RNA (shRNA) sequences that target HCF1 (shHCF1: 5′-CCGGGTAATGGTGACACACTATTTCCTCGAGGAAATAGTGTGTCACCATTACTTTTTG-3′) and HCF2 (shHCF2: 5′- CCGGTCCCACACAGCTGTTATATATCTCGAGATATATAACAGCTGTGTGGGATTTTTG-3′), respectively, were selected from seven different shRNAs that target distinct regions of each target gene and then cloned into the lentiviral expression vector pLKO.1 (16). As a negative control, a non-target scrambled sequence (5′-CCGGCCTAAGGTTAAGTCGCCCTCGCTCGAGCGAGGGCGACTTAACCTTAGGTTTTTG-3′) was also inserted into the same vector. The lentivirus production and infection of target cells were conducted as described previously (14).

### Generation of HeLa-based AFF4 knockout (KO) cells

The procedure for using CRISPR-Cas9 to knock out the AFF4-coding gene in HeLa cells was described previously (17). The plasmid vector pSpCas9 (BB)-2A-Puro (PX459) that expresses Cas9 and sgRNA was from Addgene (plasmid #48139). The sgRNA was transcribed from 5′-CACCGAGAACGGGAAAGGCGGAATC-3′ that was inserted into the vector. The positive KO clone was identified by Sanger sequencing of the genomic amplicons obtained with the TA Cloning Kit (Life Technologies), and the loss of expression of AFF4 was verified by Western blotting.

### Reverse transcription followed by quantitative real-time PCR (RT-qPCR) analysis

Total RNA was extracted with the RNA exaction Kit (Qiagen) and reverse transcribed using random hexamer primers (Life Technologies). The cDNA was amplified following the procedure for quantitative real-time PCR analysis as described previously (17). The PCR primers were designed with Integrated DNA Technologies’ Primer Quest, and their sequences listed in Supplemental Table S1. All reactions were performed in triplicates to ensure reproducibility. The PCR signals were normalized to those of GAPDH and displayed as relative folds of induction.

### Co-immunoprecipitation

The assay was performed as described previously (18) with minor modifications. Briefly, 600 μl nuclear extracts (NE) or whole cell extracts (WCE) were pre-cleared by incubation with 50 μl protein A-beads for 1 h at 4°C and the supernatants were incubated with 50 μl anti-HA or anti-Flag agarose beads (Sigma) for 2 h. The beads were washed twice with buffer D (20 mM HEPES–KOH [Ph7.9], 15% glycerol, 0.2 mM EDTA, 0.1% NP-40, 1 mM dithiothreitol, 1 mM phenylmethylsulfonyl fluoride) containing 0.3 M KCl and then twice with buffer D containing 0.1 M KCl. The precipitates were eluted off the beads by incubation with 0.1 M glycine (pH 2.5) and analyzed by Western blotting with the indicated antibodies.

### *In vivo* ubiquitination assay

The assay was performed as described previously (8) with some minor modifications. Briefly, at 40 h post transfection with the indicated plasmids, cells were treated with MG132 for additional 8 h. Cells were suspended in buffer A (6 M guanidine–HCl, 0.1 M Na_2_HPO_4_/NaH_2_PO_4_, and 10 mM imidazole), and frozen overnight at -80°C. The thawed cell suspension was sonicated for a total of 5 min on ice consisting of cycles of 10 s on and 20 s off at 50% power amplitude to make whole cell extracts (WCE). The WCE were then incubated with the Ni^2+^-NTA beads (QIAGEN) and the pull down products were analyzed by Western blotting with the indicated antibodies as described (8).

### ChIP-qPCR assay

The assay was performed as described previously (15) with minor modifications. Briefly, NH1 cells expressing shScramble, shHCF1 or shHCF2 were harvested and subjected to the ChIP procedure. After the diluted sheared chromatin DNA was pre-cleared with 60 μl protein A-beads for 1 h at 4°C, the supernatants were incubated with 5 μg anti-ELL2 antibody or 5 μg total rabbit IgG for each immunoprecipitation (IP). DNA samples thus purified were analyzed by quantitative PCR with the primers listed in Supplemental Table S1.

## RESULTS

### HCF1 and HCF2 are required for HIV-1 transcription and their cellular levels are insufficient for robust Tat-transactivation

**Figure 1.**
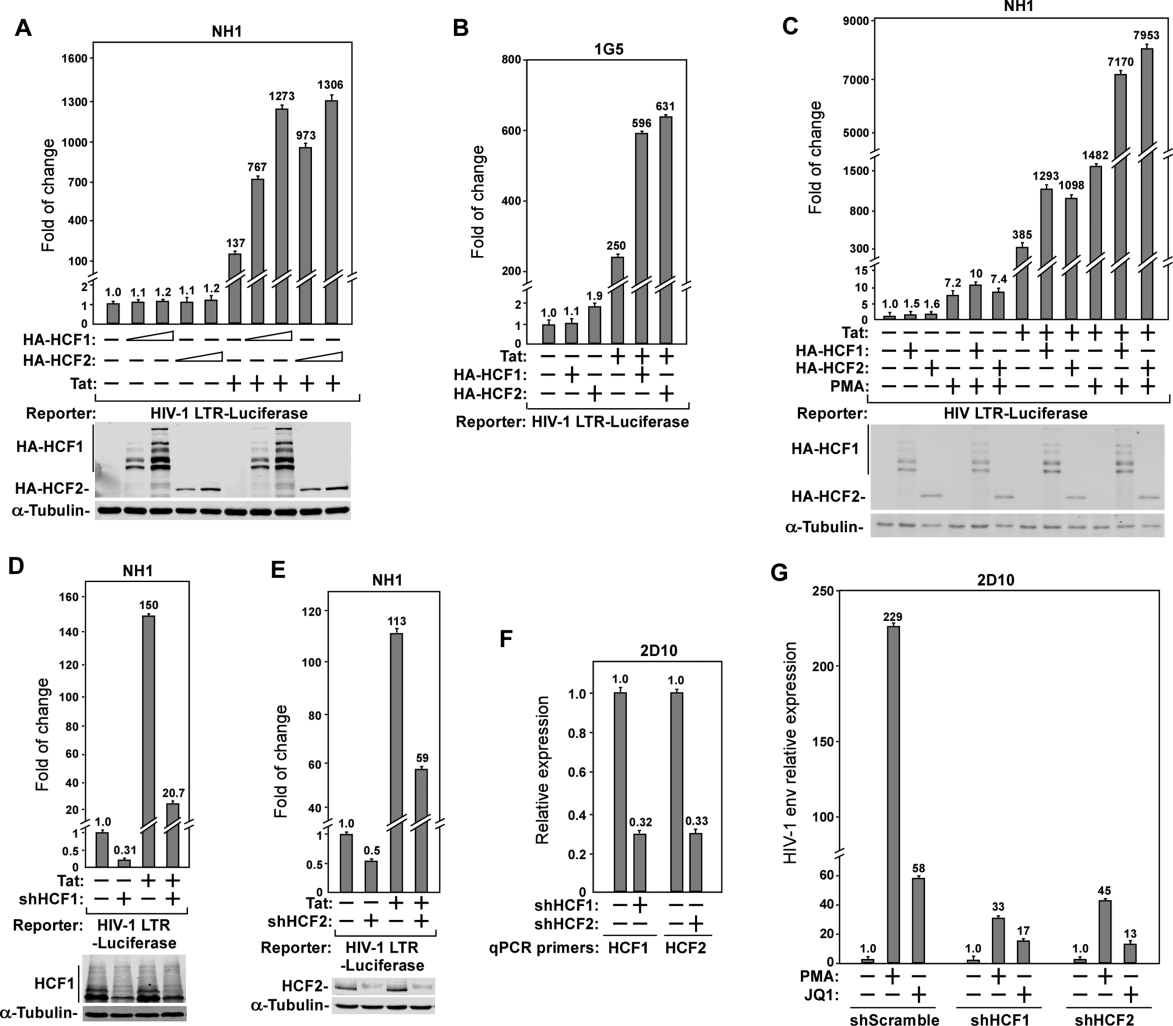
HCF1 and HCF2 are required for HIV-1 transcription and their cellular levels are insufficient for robust Tat-transactivation. (**A**–**C**) The HeLa-based NH1 cells (A and C) and Jurkat-based 1G5 cells (B), both of which contain an integrated HIV-1 LTR-luciferase reporter construct, were transfected with either an empty vector (−) or the vector expressing HA-HCF1, HA-HCF2 or/and Tat as indicated. At 24 h post-transfection, the cells in C were either untreated (−) or treated with PMA for another 24 h. At 48 h post-transfection, whole cell extracts (WCEs) were prepared and luciferase activities were measured and plotted, with the activities present in lane 1 in each panel set to 1.0. The indicated proteins in WCE were examined by Western blotting (WB) in A and C. (**D, E**) NH1 cells were transfected with an empty (−) or Tat-expressing vector and also vectors expressing shHCF1 (D), shHCF2 (E), or a non-specific scrambled sequence (−). Luciferase activities in WCE were examined and analyzed as in A. (**F**) The shRNA-induced knockdown efficiency of HCF1 or HCF2 mRNA levels in Jurkat-based 2D10 cells was examined by RT-qPCR, divided by those of GAPDH, and normalized to the signals in cells expressing the scrambled sequence, which is set to 1.0. (**G**) 2D10 cells expressing the indicated shRNA were treated with DMSO (−), PMA or JQ1 for 24 h. The HIV-1 *env* mRNA levels were measured by RT-qPCR and analyzed as in F. Error bars in all panels represent mean ± SD from three separate measurements.

Recent evidence has indicated a role of HCF1 in regulating transcriptional elongation of the HSV IE genes through interacting with the human SEC (13). Since it is well known that the SEC is also important for HIV-1 Tat-transactivation (2–5), we decided to investigate whether HCF1 also controls this process, and if so, whether its interaction with the SEC may be involved. To this end, we first examined the impact of overexpressing HCF1 or its homolog HCF2 on HIV-1 LTR activity in HeLa-based NH1 cells containing an integrated HIV-1 LTR-luciferase reporter construct (3). The data indicate that both HCF1 and HCF2 dose-dependently activated the HIV-1 LTR-driven luciferase expression only when the viral Tat protein was also co-expressed in the cells (Figure 1A). In the absence of Tat, neither HCF1 nor HCF2 had much effect on basal LTR activity. Notably, the overexpressed HCF1 but not HCF2 displayed the signature glycosylation-dependent cleavage pattern during maturation as reported previously (19) (Figure 1A). A similar effect of the overexpressed HCF1/2 on basal and Tat-activated HIV-1 transcription was also obtained in the Jurkat-based IG5 cell line that harbors an integrated HIV-1 LTR-luciferase reporter gene (Figure 1B).

Since HIV-1 transcription can also be stimulated in a Tat-independent manner by the PKC activator PMA (20), we overexpressed HCF1 or HCF2 in PMA-treated NH1 cells and found that the overexpression produced no or only very minor effect on HIV-1 transcription (Figure 1C). In contrast, a much more pronounced effect of HCF1/2 was detected in both PMA-treated and untreated cells that expressed Tat (Figure 1C). Together, these results suggest that the endogenous HCF1/2 levels were rate-limiting for Tat-dependent but not basal or PMA-induced HIV-1 transcription.

Next, we investigated the effect of silencing the expression of HCF1/2 with specific shRNAs on HIV-1 transcription. The knockdown (KD) in NH1 cells significantly decreased both basal and Tat-dependent HIV-1 LTR activity (Figure 1D and E). Furthermore, the requirement of HCF1/2 for HIV-1 transcription was also observed in Jurkat-based 2D10 cells, a frequently used post-integration latency model containing the d2EGFP-coding sequence in place of the viral *nef* gene in the proviral genome (21). While the latency-reversing agents (LRAs) PMA and JQ1 efficiently induced the HIV *env* mRNA production as determined by RT-qPCR in 2D10 cells expressing a scrambled shRNA sequence, the KD of HCF1 or HCF2 (Figure 1F) with the specific shRNA markedly reduced the abilities of the two LRAs to activate *env* expression (Figure 1G). Taken together, the above results are consistent with the notion that HCF1 and HCF2 are required for HIV-1 transcription and that their endogenous levels are insufficient for robust Tat-dependent HIV-1 transactivation.

### HCF1 and HCF2 stabilize ELL2 and promote formation of ELL2-containing SEC

HIV-1 transcriptional elongation, especially the Tat-activated process, requires the multi-subunit SEC that contains in one complex two different elongation stimulatory factors, ELL2 and P-TEFb, which can synergistically support Tat-transactivation (3,4,17,18). Among all the SEC subunits, ELL2 is very unstable and uniquely regulated at the level of protein stability by the E3 ubiquitin ligase Siah1 (8,22,23). In light of these observations, we decided to investigate whether HCF1/2 may promote HIV-1 transcription through affecting the expression of ELL2.

**Figure 2.**
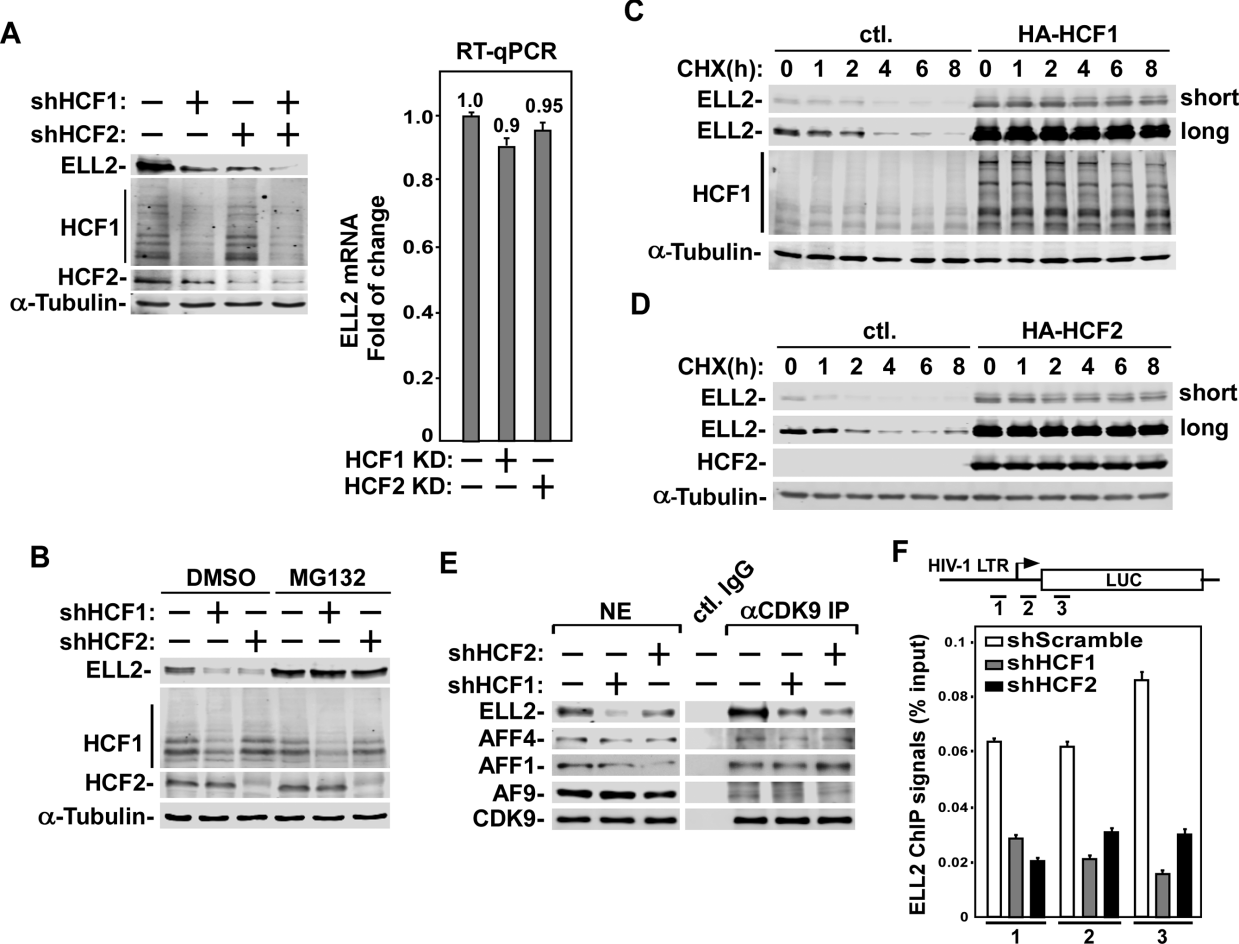
HCF1/2 stabilize ELL2 and promote formation of ELL2-containing SEC. (**A**) Left: Whole cell extracts (WCE) prepared from 293T cells expressing the indicated specific shRNAs or a scrambled sequence (−) were analyzed by Western blotting (WB) for the indicated proteins. Right: RNAs isolated from cells examined in the first three lanes in A were analyzed by RT-qPCR for the ELL2 mRNA levels. The signals were divided by those of GAPDH mRNA, and normalized to the signal in cells expressing the scrambled sequence (−), which is set to 1.0. Error bars represent mean ± SD from three separate measurements. (**B**) 293T cells expressing the indicated shRNAs were treated with DMSO or MG132 for 6 h. WCE were prepared and analyzed by WB as in A. (**C**, **D**) 293T cells were transfected with an empty vector (ctl.) or the vector expressing HA-HCF1 (C) or HA-HCF2 (D) for 40 hr. The cells were then treated with cycloheximide (CHX) and harvested at different time points as indicated. WCE were prepared and analyzed by WB. (**E**) Nuclear extracts (NE) prepared from cells expressing the indicated shRNAs or a scrambled sequence (−) are subjected to immunoprecipitation (IP) with either the anti-CDK9 polyclonal antibodies or rabbit total IgG (ctl. IgG). Both NE and the IP products were analyzed by WB for the proteins marked on the left. (**F**) NH1 cells expressing the indicated shRNA sequences were subjected to chromatin immunoprecipitation (ChIP) assay with anti-ELL2 antibody. The ELL2 occupancy on HIV-1 chromatin template at three separate locations was analyzed by qPCR using the primer pairs labeled as 1, 2 and 3 and displayed as percentages of input DNA. Error bars represent mean ± SD from three separate measurements.

Indeed, in HCF1 or HCF2 KD cells, the protein but not mRNA level of ELL2 significantly decreased (Figure 2A). The simultaneous KD of both HCF1 and 2 further decreased the ELL2 protein level (Figure 2A). It is interesting to note that pre-treating cells with the proteasome inhibitor MG132 effectively reversed the HCF1/2 KD-induced decrease in ELL2 protein level (Figure 2B). These results suggest that both HCF1 and HCF2 increased the cellular ELL2 protein level likely by preventing the proteasomal degradation of ELL2.

To confirm that it is indeed the stability but not fresh synthesis of the ELL2 protein that is promoted by HCF1/2, we examined the impact of HCF1/2 expression on half-lives of ELL2 in cells that were untreated or treated with cycloheximide (CHX) for various hours to inhibit new protein synthesis. Data in Figure 2C and D indicate that the ectopic expression of either HCF1 or two significantly increased the ELL2 protein level. More importantly, in the absence of HCF1/2, the existing ELL2 level was decreased about half after 2 h of CHX treatment and further diminished upon longer treatment. In contrast, the presence of HCF1/2 drastically lengthened the ELL2 half-life and stabilized the protein even after a prolonged CHX treatment (Figure 2C and D).

Since ELL2 is an integral SEC subunit, the formation of the ELL2-containing SEC was examined after the KD of HCF1 or 2. As expected, after the KD, there was a significant decrease of the levels of ELL2 but not other SEC subunits (CDK9, AFF4, AFF1 and AF9) in both nuclear extracts (NE) as well as in anti-CDK9 immunoprecipitates (Figure 2E). The KD also produced little effect on the Tat protein level (Supplemental Figure S1). Consistent with ELL2’s role in HIV-1 transcription, the occupancy of ELL2 at three separate locations at and around the viral promoter also showed a significant decrease after the KD of HCF1 or HCF2 as revealed by our ChIP-qPCR analysis (Figure 2F). These results support the view that HCF1 and HCF2 regulate HIV-1 transcription through effectively stabilizing the ELL2 protein and promoting the ELL2-SEC formation.

### HCF1 and HCF2 antagonize Siah1 E3 ubiquitin ligase activity to stabilize Siah1 and Siah1 substrates including ELL2

**Figure 3.**
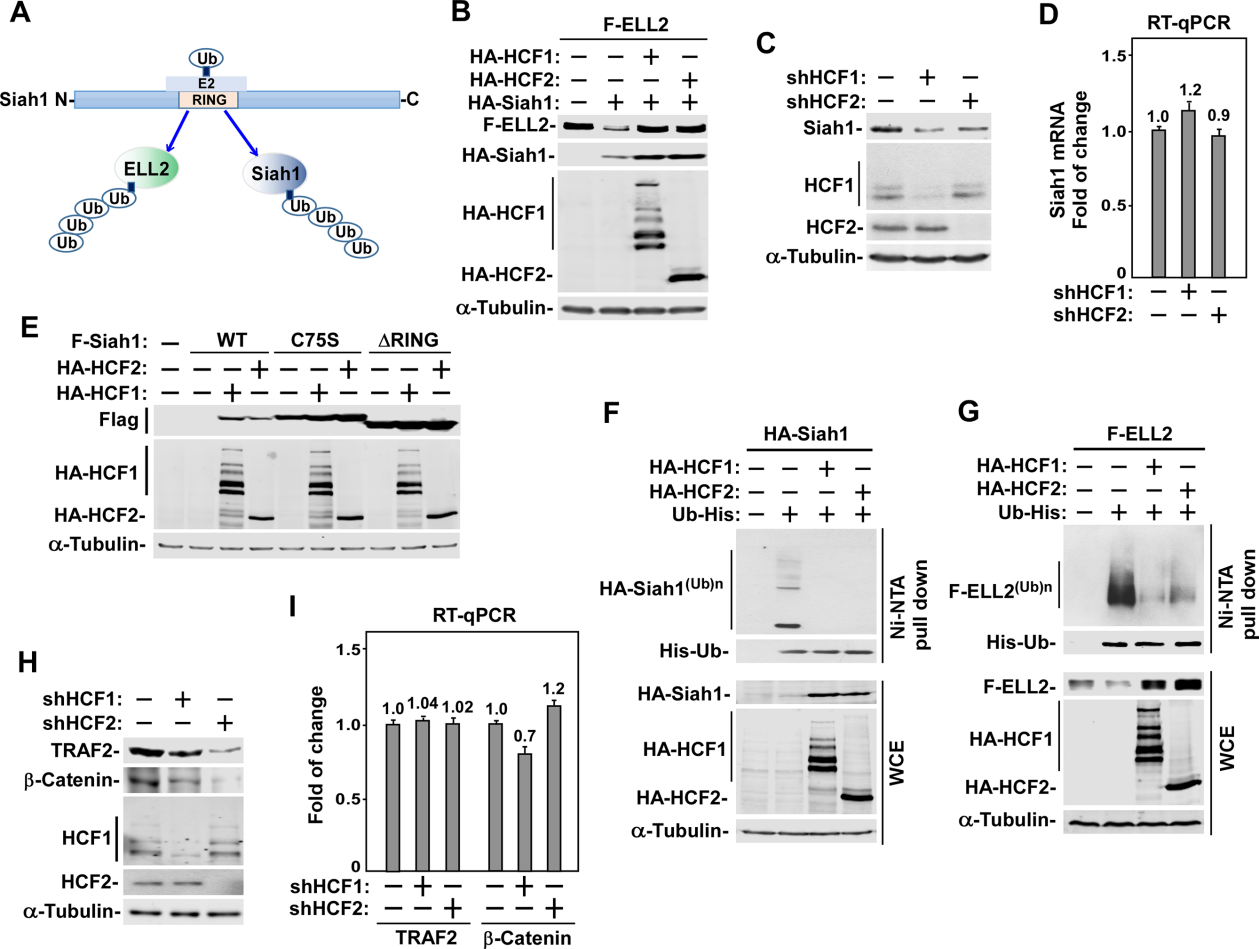
HCF1/2 antagonize Siah1’s E3 ligase activity to stabilize Siah1 and Siah1 substrates including ELL2. (**A**) A diagram showing the role of Siah1 as an E3 ubiquitin ligase for regulating the stability of ELL2 and Siah1 itself through polyubiquitination. (**B, C**) Whole cell extracts (WCE) of 293T cells transfected with plasmids expressing the indicated proteins (B), or a scrambled sequence (−), shHCF1 (HCF1 KD) or shHCF2 (HCF2 KD) (C) were analyzed by Western blotting (WB). (**D**) The Siah1 mRNA levels in cells examined in C were detected by RT-qPCR, divided by those of GAPDH, and normalized to the signal in cells expressing the scrambled sequence, which is set to 1.0. Error bars represent mean ± SD from three separate measurements. (**E, H**) WCE of 293T cells transfected with plasmids expressing the indicated proteins (E) or shRNA (H) were examined by WB for the proteins marked on the left. (**F, G**) 293T cells were transfected with plasmids expressing the indicated proteins for 42 hr and then treated with MG132 for additional 6 hr. WCE were prepared, incubated with the Ni-NTA beads, and the pull-down products analyzed by WB to detect the polyubiquitinated Siah1 (F) and ELL2 (G). (**I**) The TRAF2 and β-Catenin mRNA levels in cells analyzed in H were measured by RT-qPCR, divided by those of GAPDH, and normalized to the signals in cells expressing the scrambled sequence, which is set to 1.0. Error bars represent mean ± SD from three separate measurements.

As the E3 ubiquitin ligase for ELL2, Siah1 is responsible for ELL2’s polyubiquitination and destabilization (8). The catalytic RING domain of Siah1 can promote the transfer of ubiquitin to ELL2 and can also cause Siah1 self-ubiquitination (Figure 3A), leading to degradation of both proteins by the proteasome.

To investigate whether HCF1/2 stabilize ELL2 through antagonizing the Siah1 activity, we examined the effects of HCF1 or 2 on Siah1-mediated ELL2 degradation in cells. As expected, the co-expression of Siah1 and ELL2 caused a marked reduction in the level of ELL2 comparing to the expression of ELL2 alone (Figure 3B). More importantly, the Siah1-induced ELL2 degradation was effectively blocked when extra HCF1 or 2 was introduced into the cells. To our surprise, the Siah1 protein level also displayed an obvious increase in the presence of HCF1/2 (Figure 3B). Consistent with the notion that not only ELL2, but also its E3 ligase Siah1 depended on HCF1/2 for stability, the KD of HCF1 or 2 in cells caused a significant reduction in the endogenous level of Siah1 protein (Figure 3C) but not mRNA (Figure 3D).

Given that the stability of both ELL2 and Siah1 is controlled by Siah1 (Figure 3A), the above results suggest that HCF1/2 may directly inhibit the Siah1 activity to elevate the cellular levels of ELL2 and Siah1. To test this idea, a catalytically inactive mutant (C75S) of Siah1 as well as a deletion mutant (ΔRING) lacking the entire catalytic RING domain (aa70–109) were examined first for their responses to the introduction of HCF1 or 2 into cells. While wild-type (WT) Siah1 became dramatically stabilized by HCF1/2, the levels of C75S and ΔRING were very high to begin with (due to their lack of self polyubiquitination) and only mildly affected by the expression of HCF1/2 (Figure 3E).

A more direct demonstration of HCF1/2’s inhibition of Siah1 E3 ubiquitin ligase activity was revealed in an in vivo ubiquitination assay. When Siah1 was co-expressed in cells with HCF1 or 2 and the histidine-tagged ubiquitin (His-Ub), which can be captured by the Ni^2+^-NTA beads, an HCF1/2-induced loss of auto-polyubiquitinated Siah1 in the Ni^2+^-NTA pull-down and a concurrent stabilization of total Siah1 in whole cell extracts (WCE) were detected by Western blotting (Figure 3F).

A very similar result was also observed when Siah1 was replaced in the above assay with ELL2 (Figure 3G), indicating a direct inhibition by HCF1/2 of the Siah1 E3 ligase activity toward its exogenous substrate ELL2. It is worth noting that ELL2 is only one of the Siah1 substrates. In addition to ELL2, two additional Siah1 substrates, TRAF2 (TNF receptor-associated factor 2 (24)) and β-catenin (25), which may not have a direct role in SEC-dependent HIV-1 transcriptional control, also displayed a marked decrease in their protein but not mRNA levels upon the KD of HCF1 or 2 (Figure 3H and I). Together, these results strongly support the notion that HCF1/2 directly inhibit the Siah1 E3 ubiquitin ligase activity to stabilize the Siah1 substrates ELL2, TRAF2 and β-catenin, as well as Siah1 itself.

### HCF1/2 and ELL2 compete for binding to Siah1

How do HCF1/2 inhibit Siah1’s activity? One possibility is that HCF1/2 directly bind to Siah1 in a way that the binding interferes with the Siah1 catalytic function. To test this idea, we first examined whether HCF1/2 could block the binding of Siah1 to its substrate ELL2. The catalytically inactive Siah1 mutant C75S was used in the assay involving co-immunoprecipitation (IP) followed by Western blotting (co-IP/Western), because this point-mutation was shown to inactivate Siah1’s E3 ligase activity but not binding to the substrates (26), thus making the mutant more stable and easier to analyze than WT Siah1.

**Figure 4.**
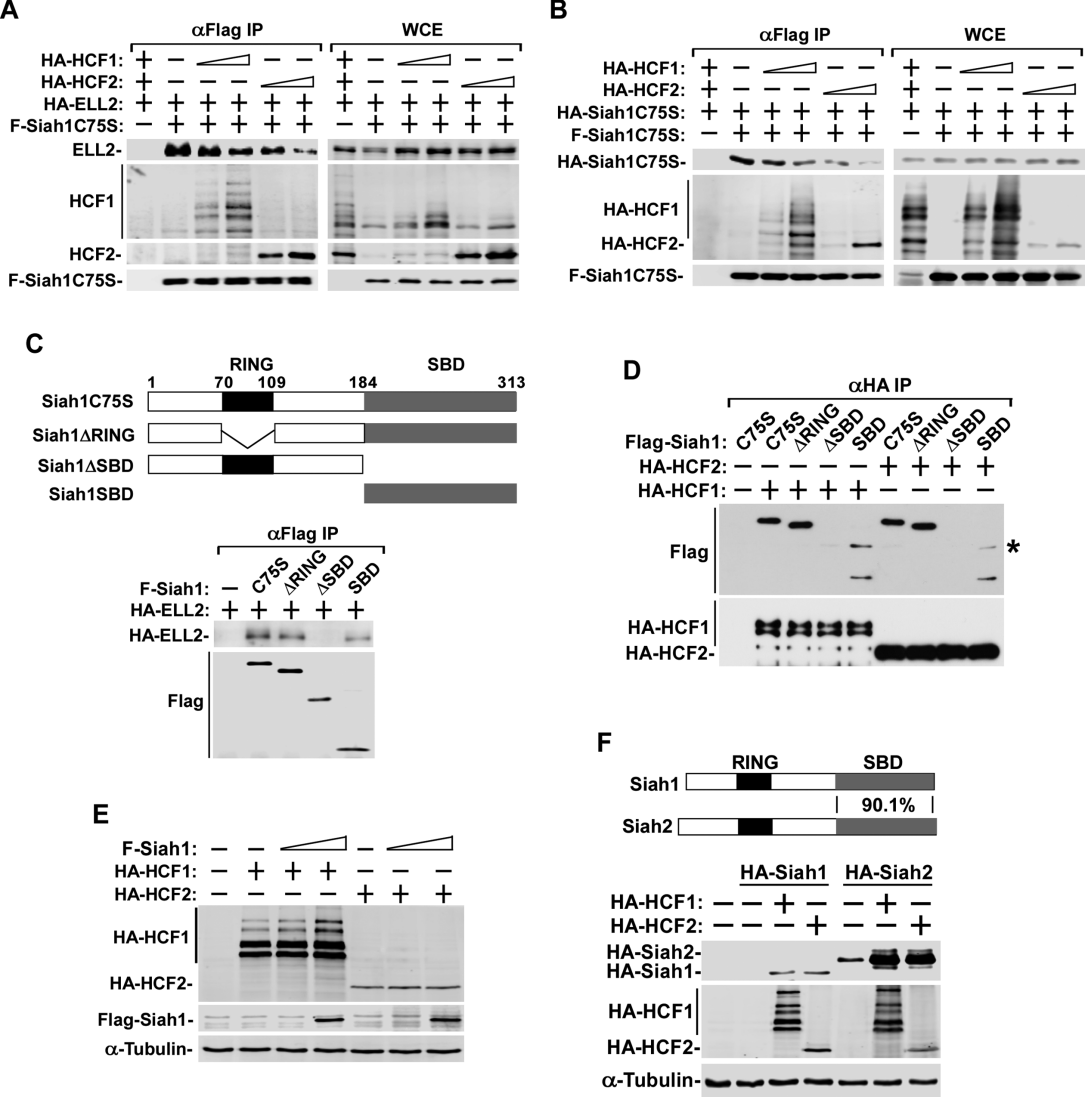
HCF1/2 bind to Siah1’s substrate-binding domain (SBD) to block ELL2-Siah1 and Siah1-Siah1 interactions to stabilize ELL2 and Siah1. (**A, B**) 293T cells were co-transfected with an empty vector (−) or the vector expressing the indicated proteins. Whole cell extracts (WCE) prepared from the cells at 48 hr post-transfection as well as the anti-Flag immuneprecipitates (αFlag IP) derived from WCE were analyzed by Western blotting (WB) for the proteins labeled on the left. (**C**) Top: A schematic diagram of domain structures of full-length Siah1 protein containing the C75S point mutation and the various deletion mutants created in the C75S background. Bottom: Plasmids expressing the indicated Flag-tagged Siah1 proteins were transfected into 293T cells and αFlag IP derived from WCE were analyzed by WB. (**D**) αHA IP from WCE of 293T cells transfected with plasmids expressing the various F-Siah1 proteins and HA-HCF1 or HA-HCF2 were analyzed by WB. A non-specific band is indicated with a star (*). (**E, F**) WCE were prepared from 293T cells co-transfected with the indicated expression constructs and examined by WB.

Indeed, the overexpression of HCF1/2 dose-dependently decreased the ELL2-Siah1 binding (Figure 4A). Interestingly, as fewer ELL2 molecules remained bound to Siah1 under these conditions, more HCF1/2 became associated with Siah1 (Figure 4A), suggesting that ELL2 and HCF1/2 competed for binding to the same Siah1 molecule. Notably, the competition between purified HA-HCF1/2 and GST-ELL2 for binding to immobilized F-Siah1C75S was also demonstrated in vitro (Supplemental Figure S2).

Since the dimerization of Siah1 is essential for Siah1’s self-ubiquitination and degradation, and in light of the earlier observations that HCF1/2 also significantly stabilized Siah1 (Figure 3B and C), the effect of HCF1/2 on Siah1 dimerization was analyzed. Similar to the effect on the ELL2-Siah1 binding, the dimerization between the HA- and Flag-tagged Siah1C75S also decreased by the overexpressed HCF1 or HCF2 in a dose-dependent manner (Figure 4B). At the same time, there was an increased interaction of HCF1/2 with the bait F-Siah1 protein. Taken together, these results suggest that HCF1/2 may disrupt the ELL2-Siah1 interaction as well as Siah1-Siah1 dimerization through competitive binding to Siah1.

### HCF1/2 bind to Siah1’s substrate-binding domain (SBD) to block ELL2-Siah1 and Siah1-Siah1 interactions

To investigate how HCF1/2 and ELL2 compete for binding to Siah1, we first set out to determine which part of Siah1 is required for the interaction with ELL2. Different Siah1 deletion mutants were created and co-expressed with ELL2 (Figure 4C). SBD, which contains just the substrate-binding domain of Siah1, and ΔSBD, a deletion mutant lacking the SBD, were again created in the C75S background in order to enhance their stability. The abilities of these two Siah1 deletion mutants, together with ΔRING, a mutant lacking the central catalytic RING domain, to pull down co-expressed ELL2 were analyzed by co-IP/Western. The data indicate that ELL2 required the C-terminal SBD of Siah1 to stably interact with Siah1 (Figure 4C).

By performing a similar co-IP/Western analysis, in which ELL2 was replaced with HCF1 or HCF2, we found that the Siah1 SBD was also required for stable interaction between Siah1 and HCF1/2 (Figure 4D). However, despite their interactions with Siah1 through SBD, the two HCF proteins did not appear to act as substrates of Siah1 as the co-expression of WT Siah1 with HCF1 or HCF2 did not cause any obvious decrease in the HCF1/2 protein levels (Figure 4E), which is quite different from the situation involving co-expression of ELL2 and Siah1 (Figure 3B). Taken together, these results indicate that ELL2 and HCF1/2 bind to the same SBD domain of Siah1. Since these binding events were mutually exclusive (Figure 4A) and that HCF1/2 are apparently not Siah1’s substrates, the ability of HCF1/2 to displace ELL2 off Siah1 explains how HCF1/2 can effectively antagonize the Siah1-induced polyubiquitination and degradation of ELL2.

Siah2 is a homolog of Siah1 and their SBDs share 90.1% overall identity. In light of this high conservation and the above observations that HCF1/2 stabilize Siah1 and its substrates by directly targeting the SBD of Siah1, it is not surprising to see that both HCF1 and HCF2 were also able to make Siah2 significantly more stable (Figure 4F). However, as reported earlier that Siah2 is not an E3 ligase for ELL2 (8), the observed stabilizing effect of HCF1/2 on ELL2 is most likely through inhibiting the function of Siah1 but not Siah2.

### AFF4 is not required for HCF1/2 to stabilize ELL2 and activate HIV-1 transcription

**Figure 5.**
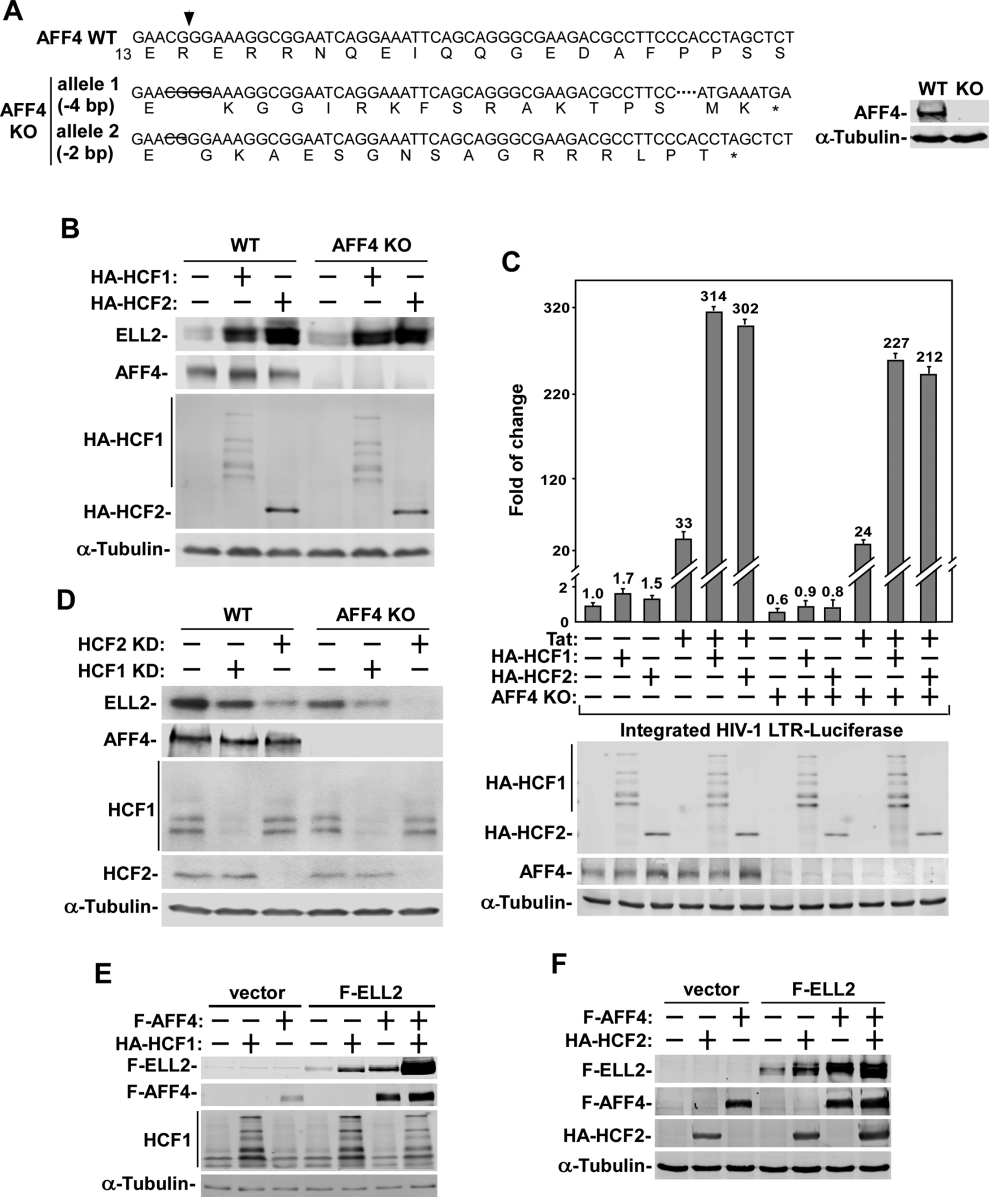
AFF4 is not required for HCF1/2 to stabilize ELL2 and activate HIV-1 transcription but can synergize with HCF1/2 to make ELL2 even more stable. (**A**) Verification of AFF4 knockout (KO) in HeLa cells. The nucleotide and predicted amino acid sequences surrounding the intended Cas9 cleavage sites (arrowhead) in wild-type AFF4 gene as well as its mutant alleles generated by CRISPR-Cas9 are shown. The deleted and omitted nucleotides in mutant alleles are indicated by strikethroughs and consecutive dots, respectively. Premature stop codons due to frame shift mutations are denoted by stars (*). Whole cell extracts (WCE) from WT and KO cells were subjected to Western blotting (WB) to confirm the loss of AFF4 expression. (**B, D**) WT and AFF4 KO cells were transfected with the plasmid expressing HA-HCF1 or HA-HCF2 (B) or plasmid expressing shHCF1 or shHCF2 (D). WCE were prepared 48 hr later and analyzed by WB for the indicated proteins. (**C**) WT and AFF4 KO cells were co-transfected with the HIV-1-LTR-luciferase reporter construct and an empty vector (−) or the vector expressing HA-HCF1, HCF2 and/or Tat as indicated. WCE were obtained 48 hr later and analyzed for their luciferase activities as well as the indicated proteins by WB. Error bars represent mean ± SD from three separate measurements. (**E, F**) WCE of 239T cells transfected with an empty vector or the vector expressing the indicated proteins were examined by WB for the proteins labeled on the left.

ELL2 is a subunit of the multi-subunit SEC complex, within which the scaffolding protein AFF4 interacts with ELL2 and other SEC subunits to hold the complex together (3,7). Recently, it has been reported that HCF1 regulates the HSV transcriptional elongation in an AFF4-dependent manner (13). To determine whether AFF4 also plays an important role in HCF1/2’s promotion of ELL2 stability and stimulation of HIV-1 transcription, we generated a HeLa-based AFF4 knockout (KO) cell line using the CRISPR-Cas9 system and verified AFF4’s loss of expression by Western blotting (Figure 5A).

The data in Figure 5B indicate that the ectopic expression of HCF1 or HCF2 in AFF4 KO cells increased the ELL2 protein level similarly as in WT cells, suggesting that unlike the effect of HCF1 on HSV transcription (13), the promotion of ELL2 stability by HCF1/2 does not depend on AFF4. Consistent with this observation, the depletion of AFF4 in the KO cells also failed to affect the ability of HCF1/2 to increase the magnitude of Tat-activated HIV-1 LTR activity, and to a smaller extent, the basal LTR function (Figure 5C). It is worth noting that the AFF4 KO had an overall minor effect on HIV-1 transcription (Figure 5C), and this agrees well with our previous demonstrations that between the two homologous AFF proteins, AFF1 and AFF4, the latter plays only a very minor role in mediating HIV-1 transcription especially Tat-transactivation (18).

### HCF1/2 and AFF4 synergistically stabilize ELL2

Although AFF4 is not required for HCF1/2 to stabilize ELL2 and promote Tat-transactivation, we have previously shown that this scaffolding protein is also capable of making ELL2 stable (8). It turns out that upon association with AFF4, ELL2 can no longer be polyubiquitinated by Siah1 and degraded by the proteasome, thus AFF4 appears to play a protective role by sequestering ELL2 away from Siah1 to maintain ELL2 stability (8). With the above demonstrations that HCF1/2 stabilize ELL2 through binding to and blocking the SBD domain of Siah1, a mechanism different from that used by AFF4, we asked whether AFF4 and HCF1/2 could work together to make ELL2 even more stable.

To answer this question, we first used specific shRNAs to knock down the expression of HCF1 or HCF2 in both WT and AFF4 KO cells and found that the KD caused the endogenous ELL2 protein levels to further decrease in the KO cells in comparison to WT cells (Figure 5D). In addition, the ability of AFF4 and HCF1/2 to cooperatively stabilize ELL2 was also revealed in an experiment involving ectopic co-expression. When ELL2 was co-expressed together with AFF4 and HCF1 or HCF2, the ELL2 protein level was elevated to a much higher level than those obtained with the co-expression of ELL2 with AFF4 or ELL2 with HCF1/2 (Figure 5E and F). Together, the above data indicate that the stability of ELL2 in vivo is promoted cooperatively by both HCF1/2 and AFF4.

## DISCUSSION

The dependence on the SEC, which packs two different classes of elongation stimulatory factors into one complex, for robust Tat-activation of HIV-1 transcription has been well-documented (2,5). Recent evidence indicates that this complex is also essential for efficient transcriptional elongation of the HSV IE genes, which employ promoter-proximal pausing and release of RNA Pol II as a key regulatory mechanism for expression (13). The involvement of the SEC in HSV IE elongation control was revealed through proteomic analysis of the proteins associated with HCF1, which had previously been shown to stimulate the initiation phase of IE transcription through binding to multiple transcription factors including the viral VP16 activator in the IE enhancer complexes (27). Although a physical interaction between HCF1 and the SEC has been established, exactly how this interaction specifically benefits Pol II elongation along the IE chromatin templates remains to be determined (13).

Mirroring the observed promotion of HSV IE elongation by HCF1, our current study has revealed a stimulatory effect of HCF1, as well as its hitherto poorly characterized homolog HCF2, on HIV-1 elongation in a manner that also involves the SEC. However, different from the above-mentioned HSV IE results, the physical HCF1-SEC interaction does not appear to play a key role in the HIV-1 elongation control. Rather, HCF1 and HCF2 work through an indirect mechanism to promote the stability of the SEC subunit ELL2, leading to formation of more ELL2-SEC and activation of HIV-1 elongation. It will be interesting to see whether this mode of action by HCF1/2 also contributes to SEC stimulation of Pol II elongation along the HSV IE genes.

Because of the tremendous importance of the Siah ubiquitin ligases in diverse biological processes ranging from cellular stress responses, gene expression control, nutrient sensing and utilization, to cellular organization and polarity, the regulation of the expression and activity of these ligases has long been a major focus of active investigations (9). Past studies have identified a number of mechanisms that control Siah1/2 at both the transcriptional and post-translational levels. For example, the expression of Siah1/2 can be modulated through transcriptional activation by certain sequence-specific transcription factors (28–33), gene amplification of the Siah2 genomic loci (34), as well as specific miRNAs (35–40). In addition, the activity of Siah1/2 can often be controlled by phosphorylation, which in some cases affects the subcellular localization of the E3s and in others their interactions of with the substrates (41–43).

Complementing these studies, our recent work has revealed a key role of poly (ADP-ribose) polymerase 1 (PARP1) in suppressing the expression of Siah1 at both the mRNA and protein levels. At the mRNA level, PARP1 coordinates with the co-repressor NCoR to suppress Siah1 transcription. At the protein level, PARP1 promotes Siah1 proteolysis through inducing PARylation-dependent ubiquitination (PARdU) of Siah1 (22).

Among the many mechanisms known to modulate Siah1/2’s expression and activity, some of which are mentioned above, it is very rare to see regulators that can accomplish this task through direct associations with the E3 ligases. In fact, there are only a few reported cases of regulators that display such a mode of action. For example, upon hypoxia induction, the p75 neurotrophin receptor is shown to bind and stabilize Siah2 by decreasing its self-ubiquitination (44). In addition, EEF1D (Eukaryotic translation elongation factor 1 delta) has been reported to directly bind to Siah1 and inhibit its catalytic activity (45). In both cases, the regulators involved, p75 and EEF1D, exert their inhibitory effects by binding to the Siah2 zinc finger and Siah1 cysteine-rich region, respectively. In light of these results, our present finding is significant in that it not only identifies HCF1/2 as novel activators of HIV-1 transcription by inhibiting the Siah ligase activity to stabilize ELL2, but also reveals the SBD of Siah1/2 as a previously unrecognized new target for HCF1/2 to exert their inhibitory effects.

## Supplementary Material

gkaa461_Supplemental_Files

## Notes

Present address: Jun Wu, School of Pharmaceutical Sciences, Xiamen University, Xiamen, Fujian 361005, China.
