# Supplementary material for: Host cell factors stimulate HIV-1 transcription by antagonizing substrate-binding function of Siah1 ubiquitin ligase to stabilize transcription elongation factor ELL2
Source: Nucleic Acids Res. 2020 Jun 1;48(13):7321–32. doi: 10.1093/nar/gkaa461 (PMC7367184; doi:10.1093/nar/gkaa461)
Supplement: gkaa461_Supplemental_Files [file gkaa461_supplemental_files.zip › Supplementary Material.docx]

| Gene | qPCR primers |
| --- | --- |
| *HCF-1* | F-GGCACCGTCCCTGACTATAA  R-GATAATCTTGCCGGAGGTCA |
| *HCF-2* | F-TATTTGGGGGAATGGTTGAA  R-GAATCTTCGCTTTCGTTTGC |
| *ELL2* | F-AAGGCCTACAAGAAACCGGA  R-AGGCCAGTCTCTTTGAAGCT |
| *Env* | F-GAGACAGAGACAGATCCATTCG  R-CCAGAAGTTCCACAATCCTCG |
| *Siah1* | F-TGTTTGTAGCAACTGTCGCC  R-AGCCACTTTCTCCATAGCCA |
| *GAPDH* | F-GAAGGTGAAGGTCGGAGTC  R-GAAGATGGTGATGGGATTTC |
| *TRAF2* | F-CGGCCACTTTTGAGAACATT  R-CCAAGACCTTCTGCTCCAAG |
| *β-Catenin* | F-GAAACGGCTTTCAGTTGAGC  R-CTGGCCATATCCACCAGAGT |
| *Promoter* (position 1) | 1. TCCGCTGGGGACTTTCCA   R-GTACAGGCAAAAAGCAGCTGC |
| *TAR*  (position 2) | F-TGCTTTTTGCCTGTACTGGGT  R-CAGTACCGGAATGCCAAGCTT |
| *Nascent* (position 3) | F-TCTGGCTAACTAGGGAACCCA  R- CGCCGGGCCTTTCTTTATGT |

**Supplemental Table S1；The primers for qPCR**


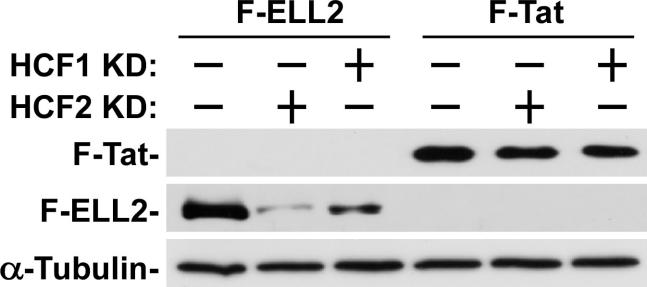


**Supplemental Figure S1. KD of HCF1 or HCF2 reduces the protein levels of ELL2 but not Tat.** 293T cells expressing the HCF1- or HCF2-specific shRNA (HCF1 KD or HCF2 KD) or a scrambled sequence (-) were transfected with 2 μg plasmid expressing either F-ELL2 or F-Tat. Whole cell extracts prepared from the cells were analyzed by Western blotting for the indicated proteins.

*
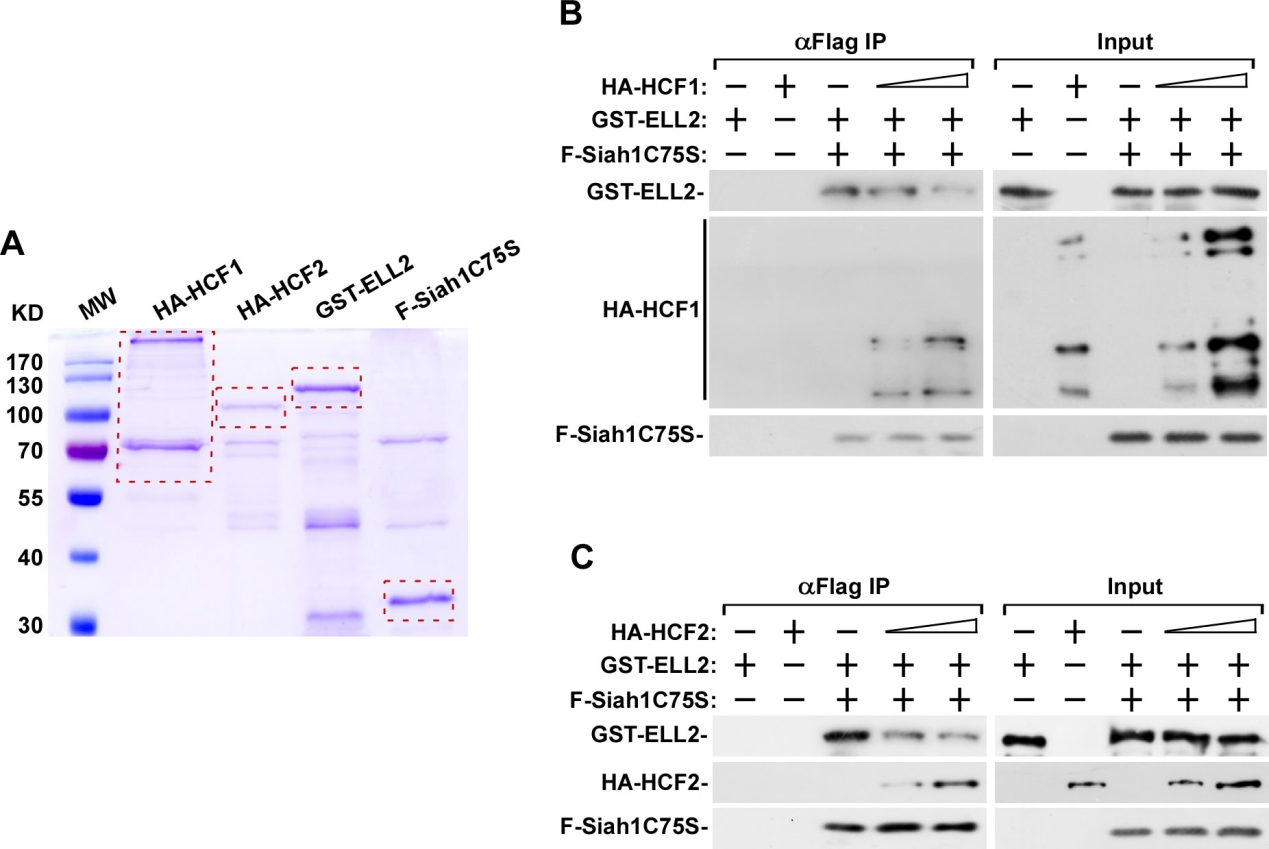
*

**Supplemental Figure S2. HCF1 and HCF2 compete with ELL2 for binding to Siah1 in vitro. (A)** Purified proteins used in the binding assay were analyzed by SDS-PAGE followed by Coomassie blue staining. HA-HCF1/2 were affinity-purified from transfected 293T cells by anti-HA immunoprecipitation (IP). After washing with buffer D1.0 containing 1M KCl, the precipitated protein was eluted off the beads with HA peptide. GST-ELL2 was expressed in and purified from *E. coli*, and F-Siah1C75S was affinity-purified by anti-Flag IP from transfected cells and kept on the Flag beads after washing with D1.0. For HA-HCF1, the dashed box contains the glycosylation-dependent cleavage products. For the rest of the proteins, the dashed boxes denote the full-length products. **(B, C)** The proteins bound to immobilized F-Siah1C75S and the input proteins added into the binding reactions were analyzed by Western blotting for the proteins labeled on the left.
