# Supplementary figures and images for: Host cell factors stimulate HIV-1 transcription by antagonizing substrate-binding function of Siah1 ubiquitin ligase to stabilize transcription elongation factor ELL2
Source: Nucleic Acids Res. 2020 Jun 1;48(13):7321–32. doi: 10.1093/nar/gkaa461 (PMC7367184; doi:10.1093/nar/gkaa461)

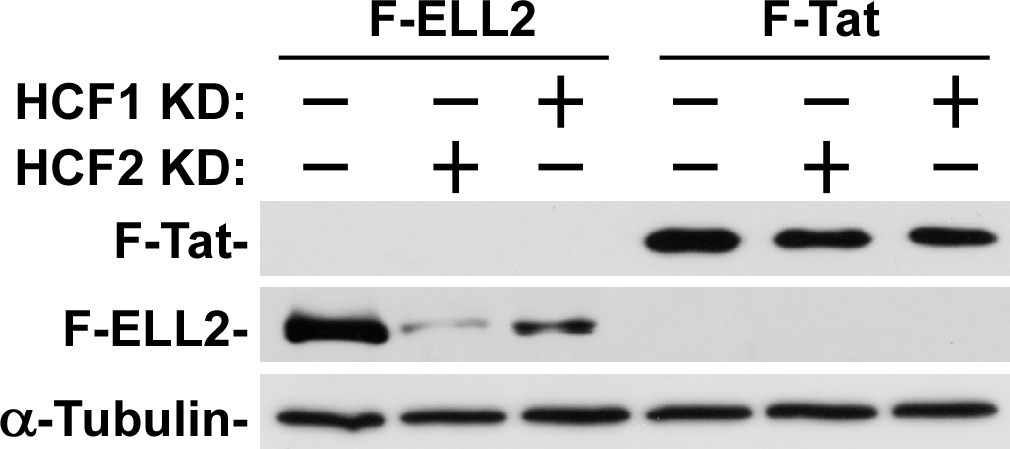

Supplement: gkaa461_Supplemental_Files [file gkaa461_supplemental_files.zip › Supplemental_Fig.S1.tif]

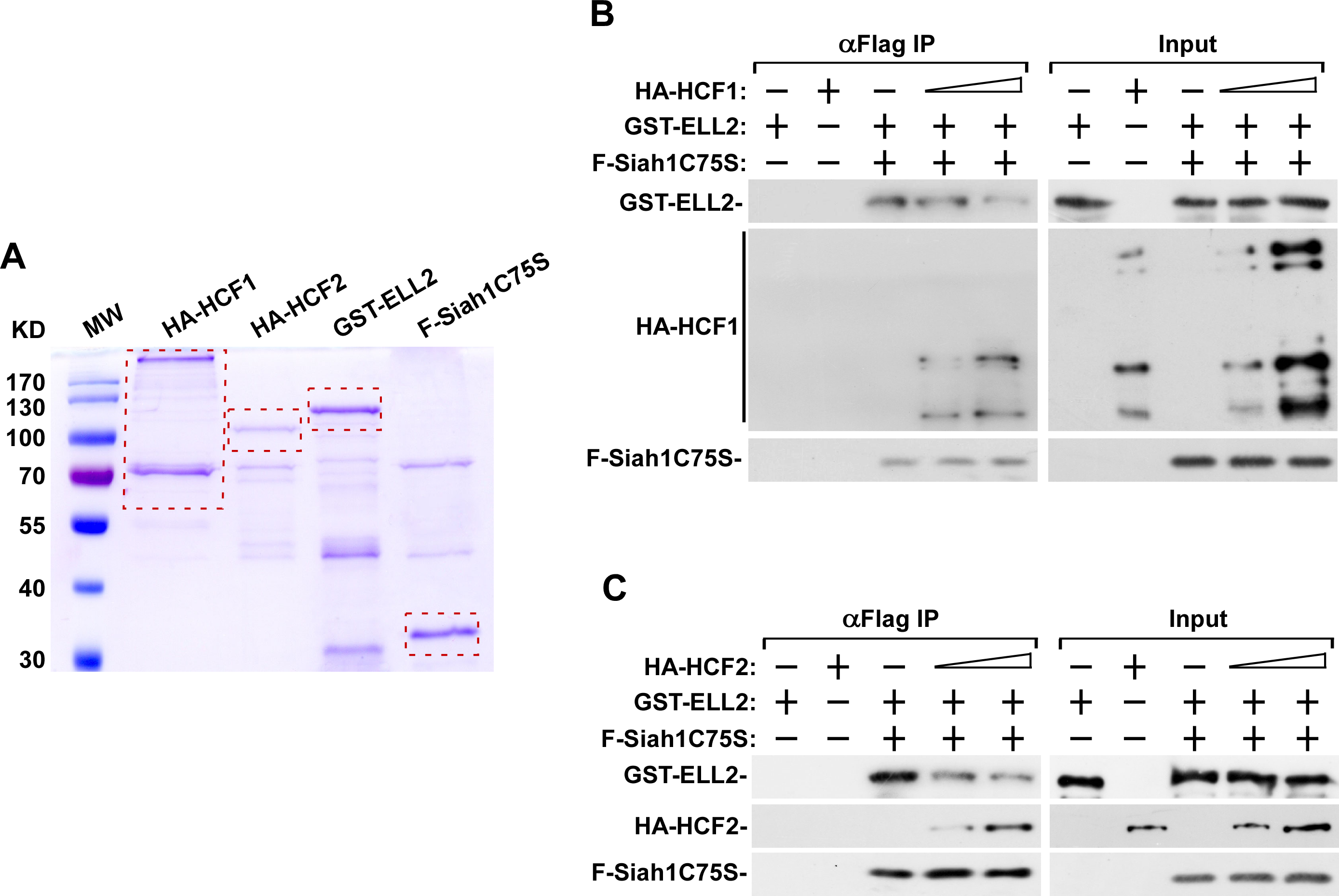

Supplement: gkaa461_Supplemental_Files [file gkaa461_supplemental_files.zip › Supplemental_Fig.S2.tif]
